# Oral Liquid l-Thyroxine (l-T4) May Be Better Absorbed Compared to l-T4 Tablets Following Bariatric Surgery

**DOI:** 10.1007/s11695-013-1015-y

**Published:** 2013-07-04

**Authors:** Ilenia Pirola, Anna M. Formenti, Elena Gandossi, Francesco Mittempergher, Claudio Casella, Barbara Agosti, Carlo Cappelli

**Affiliations:** 1 Department of Clinical and Experimental Sciences, Endocrine and Metabolic Unit, Clinica Medica, University of Brescia, 1 Medicina Spedali Civili di Brescia, Piazzale Spedali Civili n1, 25100 Brescia, Italy; 2 Department of Department of Molecular and Translational Medicine, 1st Division of General Surgery, University of Brescia, Brescia, Italy; 3 Diabetic Unit, Spedali Civili di Brescia, Place Spedali Civili 1, 25100 Brescia, Italy

**Keywords:** Severe obesity, Roux-en-Y gastric bypass surgery, l-thyroxine, Liquid formulation, Absorption

## Abstract

Drug malabsorption is a potential concern after bariatric surgery. We present four case reports of hypothyroid patients who were well replaced with thyroxine tablets to euthyroid thyrotropin (TSH) levels prior to Roux-en-Y gastric bypass surgery. These patients developed elevated TSH levels after the surgery, the TSH responded reversibly to switching from treatment with oral tablets to a liquid formulation.

## Introduction

Global demand for bariatric surgery has increased dramatically in the past decade [1]. This procedure refers to various surgical techniques employed for achieving weight loss.

Drug malabsorption is a potential postsurgical concern, particularly after diversionary procedures, for many reasons. Nearly all oral agents are maximally absorbed in the small intestine, which is bypassed in several bariatric procedures. Delayed gastric emptying, diminished opportunity for mucosal exposure, and changes in drug dissolution and solubility resulting from alterations in intestinal pH are additional factors that may potentially impair drug absorption [2, 3].

Levothyroxine (l-T4) is an effective replacement therapy for patients with hypothyroidism or suppressive therapy after surgical removal of thyroid cancer [4, 5]. Absorption of l-T4 is reduced when it is ingested concomitantly with other drugs or with coffee [6] and also when gastric pH is altered by autoimmune gastritis or by infection with *Helicobacter pylori* [7, 8]. Evidence for diminished l-T4 absorption has been reported in patients after bariatric surgery [3, 9, 10].

We observed an increase in serum thyrotropin (TSH) levels after bariatric surgery in a previously euthyroid patient receiving l-T4 in tablet form. This condition was reversibly resolved by switching to the same dose in a liquid oral formulation. We subsequently identified three additional stable hypothyroid patients submitted to bariatric surgery, who normalised serum TSH after switching from tablets to the oral liquid formulation.

## Patients and Methods

Serum concentrations of free thyroxine (fT4; normal range, 8.0–19.0 pg/mL; analytical sensitivity, 1 pg/mL; intra and inter-assay coefficient of variation, 2.4 and 6.8 %, respectively), free triiodothyronine (fT3; normal range, 2.4–4.7; analytical sensitivity, 0.35 pg/mL; intra and inter-assay coefficient of variation, 4.6 and 6.5 %, respectively), and TSH (normal range, 0.4–4.5 mIU/L; analytical sensitivity, 0.004 mlU/L; intra and inter-assay coefficient of variation, 2.5 and 5.7 %, respectively) were measured by means of immunochemoluminiscent assays using an automated analyser (Immulite 2000, DPC Cirrus, Los Angeles, CA, USA) employing commercial kits (Diagnostic Products Corporation, Los Angeles, CA, USA).

## Case Reports

Patient 1 was a 64-year-old obese Caucasian woman (BMI, 44.6 kg/m^2^) who was receiving stable substitutive therapy for hypothyroidism due to Hashimoto’s thyroiditis. In November 2009, she underwent bariatric surgery (Roux-en-Y gastric bypass). She was euthyroid receiving 200 μg l-T4 daily in tablet form. One year after surgery, she had lost 35 kg, but her thyroid hormone profile revealed subclinical hypothyroidism. We changed the formulation from oral tablets to liquid, maintaining the same dosage. After 2 months, her TSH levels were in the normal range and she felt better. To confirm the presumed relationship between oral formulation and TSH normalisation, l-T4 was re-administered at the same dosage in tablet form. Once again, serum TSH increased (Table 1). The patient verified medication compliance.

**Table 1:** Thyroid parameters measured at the indicated times in four patients who underwent bariatric surgery between 2009 and 2011. Patients were receiving oral l-T4 in either tablet or liquid form as indicated

	Before surgery l-T4 in tablet form				12 Months after surgery l-T4 in tablet form				14 Months after surgeryL-T4 in liquid form				17 Months after surgery l-T4 in tablet form			
Patient	l-T4 (μg)	TSH	fT4	fT3	l-T4 (μg)	TSH	fT4	fT3	l-T4 (μg)	TSH	fT4	fT3	l-T4 (μg)	TSH	fT4	fT3
1	200	4.2	12.7	3.1	200	18.1	10.4	2.9	200	1.5	12.9	3.8	200	36.7	9.8	3.0
2	150	3.1	12.9	3.3	150	12.1	10.2	3.2	150	1.9	13.5	4.0	150	24.7	10.4	3.2
3	200	3.9	11.7	3.7	200	20.4	10.2	3.3	200	0.6	13.5	3.2	200	17.7	10.2	3.1
4	150	3.6	10.9	3.2	150	17.2	11.0	2.8	150	2.4	11.9	3.2	150	15.3	10.1	3.1

*l* *-T4* levothyroxine, *TSH* thyrotropin, *fT4* free tyroxine, *fT3* free triiodothyronin

Patient 2 was a 33-year-old obese Caucasian woman (BMI, 45.8 kg/m^2^) who was receiving stable replacement therapy after a thyroidectomy for benign multinodular goitre. In September 2010, she underwent Roux-en-Y gastric bypass. She had been euthyroid receiving 150 μg daily of l-T4 tablets. One year after surgery, she had lost 41 kg. At that time, her serum TSH was 12.1 mIU/L, fT4 was 10.2 pg/mL, and fT3 was 3.2 pg/mL. Based on our previous experience with patient 1, we decided to switch from tablets to an oral liquid formulation, maintaining the same dosage also in this case. Euthyroidism was restored after 2 months. Once again, when l-T4 was re-administered in tablet form at the same dosage, serum TSH levels increased (Table 1).

Patient 3 was a 29-year-old obese Caucasian woman (BMI, 45.1 kg/m^2^) who was receiving stable replacement therapy for Hashimoto’s thyroiditis. In December 2010, she underwent Roux-en-Y gastric bypass surgery. She was euthyroid receiving 200 μg of l-T4 daily in tablet form. One year after surgery, she had lost 39 kg; however, her thyroid profile had worsened. Also in this case, we introduced an oral liquid formulation, maintaining the same dosage, and obtained a rapid normalisation of TSH levels. Once again, when l-T4 was re-administered in tablet form at the same dosage, serum TSH increased (Table 1).

Patient 4 was a 39-year-old obese Caucasian woman (BMI, 43.9 kg/m^2^) who was on stable replacement therapy for Hashimoto’s thyroiditis. In January 2011, she underwent Roux-en-Y gastric bypass surgery. She was euthyroid receiving 150 μg of l-T4 daily in tablet form. One year after surgery, she had lost 34 kg. At that time, her serum TSH was 17.2 mIU/L, fT4 was 11.0 pg/mL, and fT3 was 2.8 pg/mL. We replaced her l-T4 tablets with an oral liquid formulation, maintaining the same dosage. Euthyroidism was restored after 2 months. Once again, re-administration of l-T4 at the same dosage in tablet form resulted in an increase in serum TSH (Table 1).

## Discussion

All four of the hypothyroid patients described in this report had a substantial decrease in serum TSH levels after switching from treatment with thyroxine tablets to an oral liquid formulation.

After oral administration, approximately 60–90 % of an l-T4 dose is absorbed within 3 h of ingestion. A majority of the absorption takes place in the jejunum and ileum [11]. Absorption is maximal when administered on an empty stomach, reflecting the importance of gastric acidity in the process. For this reason, l-T4 is usually taken with water in the morning before breakfast [12]. Benvenga et al., clearly showed that ingesting l-T4 treatment with coffee, or with water followed by coffee within a few minutes, results in poor TSH response in many patients [13]. Moreover, concomitant assumption of a number of drugs and other substances can reduce l-T4 absorption; these include aluminium hydroxide [14], sucrafate [15], ferrous sulphate [16], cholestyramine [17], calcium carbonate [18] and fibre supplements [19].

Drug malabsorption is a potential concern after bariatric surgery, particularly after diversionary procedures. All of our patients underwent Roux-en-Y gastric bypass (RYGB), which bypasses almost the entire stomach. It consisted of transecting the upper stomach, creating a small proximal gastric pouch, which is then anastomosed to the proximal jejunum. A recent review by Padwall et al. points out that highly lipophilic drugs and/or those that undergo enterohepatic recirculation, such as levothyroxine, have the greatest potential for malabsorption after bariatric surgery [3].

Our observations are at odds with the findings of Rubio et al., who compared the mean absorption of l-T4 tablets in 15 obese patient candidates for RYGB bypass surgery, with that of 15 patients who had undergone such surgery 2–3 months previously and found no significant difference between groups regarding l-T4 absorbance [6]. There are some differences between our observations and this study. Patients in this study had undergone surgery 2–3 months prior to assessment, while in all four of our patients, the aberrant thyroid parameters were apparent 12 months after surgery. In addition, three of our four patients were receiving l-T4 for hypothyroidism due to Hashimoto’s thyroiditis, while all 15 patients who underwent surgery in the study by Rubio et al. had thyroid nodular disease. Indeed, the authors pointed to altered l-T4 absorption in some patients as a reason to monitor thyroid function parameters carefully after RYGB in patients on stable l-T4 treatment [6]. In agreement with Azizi et al. and Bevan et al. [9, 10], the serum TSH levels in our patients increased after bypass surgery, suggesting a malabsorption of l-T4. Interestingly, TSH levels normalised after switching from tablets to liquid oral formulation, and both fT4 and fT3 increased into the normal range. Consistent with a hypothesis of reduced absorption of tablets, TSH levels returned to high-normal 2 months after re-introduction of tablets.

To our knowledge, this is the first report showing reversible normalisation of serum TSH levels in patients submitted to bariatric surgery who received l-T4 in tablet form after switching to an oral liquid formulation. At present, we do not have an explanation for this observation. The fact that the change from tablets to oral formulation normalised serum TSH levels, and that switching back to tablets caused thyrotropin levels to worsen, leads us believe that absorption of thyroxine is greater with oral liquid formulations in our patients after bariatric surgery.

Patients with impaired acid secretion require a higher dose of thyroxine, suggesting that normal gastric acid secretion is necessary for effective absorption of l-T4 [7]. Drug dissolution and solubility may be altered by restrictive procedures that increase gastric pH in the newly created stomach pouch. This may occur in gastric bypass or gastroplasty, in which the new gastric pouch is partitioned from acid-producing cells in the remaining stomach [3]. Recently, Saraceno et al. have shown that the liquid formulation of l-T4 is an extremely effective means to circumvent the problem of incomplete absorption of the l-T4 of caused by proton pump inhibitor-induced increases in gastric pH [20]. It is conceivable that this formulation could also circumvent the pH alteration resulting from gastric bypass.

Another hypothesis is that the presence of alcohol (only in the liquid formulation) could play a key role in thyroxine absorption. Indeed, oral mucosal drug delivery is known as an alternative method for systemic drug delivery that offers several advantages over both injectable and enteral methods [21]. Because the oral mucosa is highly vascularised, drugs that are absorbed through the oral mucosa directly enter the systemic circulation, bypassing the gastrointestinal tract [21]. Consistent with this hypothesis, we would expect to see more rapid pharmacokinetics if oral liquid thyroxine can be absorbed by oral mucosa. Further studies are needed to clarify this intriguing point.

Finally, it is important to underline that liquid T4 formulation (Tirosint® fiala monouso, IBSA Farmaceutici Italia) is today available only in Italy, and that at the dosage of 100 μg daily is more expensive that tablets (0.06 than 0.33 Euro, respectively, daily).

In summary, we report four patients submitted to bariatric surgery, in whom oral liquid l-thyroxine induced a reversible normalisation of thyrotropin levels. It is likely that patients affected by condition that impair l-T4 absorption (e.g., bariatric surgery) could benefit from a liquid formulation.
